# Histological Correlation for Radiofrequency and Microwave Ablation in the Local Control of Hepatocellular Carcinoma (HCC) before Liver Transplantation: A Comprehensive Review

**DOI:** 10.3390/cancers13010104

**Published:** 2020-12-31

**Authors:** Peiman Habibollahi, Rahul A. Sheth, Erik N. K. Cressman

**Affiliations:** Department of Interventional Radiology, Division of Diagnostic Imaging, MD Anderson Cancer Center, Houston, TX 77030, USA; phabibollahi@mdanderson.org (P.H.); rasheth@mdanderson.org (R.A.S.)

**Keywords:** hepatocellular carcinoma, thermal ablation, radiofrequency ablation, microwave ablation, histopathology

## Abstract

**Simple Summary:**

Liver cancer is a growing problem around the world. Drugs for liver cancer have limited effect, there are not enough donors for liver transplants and many patients are not candidates for surgery to remove the tumor. In many of these cases, hyperthermia can destroy the tumor in situ with minimally invasive methods such as radiofrequency or microwave ablation. In this paper we review the literature evaluating success rates for complete ablation as judged by actual examination of treated tumors that were removed when patients received a liver transplant. While notable successes can be achieved with ablation, the published studies indicate both that complete treatment is not as common as thought and that imaging methods such as computed tomography and magnetic resonance scans do not completely identify residual cancer. There is therefore an important opportunity for improvement in the treatment of this disease.

**Abstract:**

Radiofrequency ablation (RFA) and microwave ablation (MWA) are the most widely studied and applied ablation techniques for treating primary and secondary liver tumors. These techniques are considered curative for small hepatic tumors, with post-ablation outcomes most commonly assessed by an imaging follow up. However, there is increasing evidence of a discrepancy between radiological and pathological findings when ablated lesions are evaluated following liver resection or liver transplantation. A comprehensive review of the available literature reporting the complete pathological response (cPR) following RFA and MWA was performed to estimate the success rate and identify the factors associated with treatment failure. Following RFA, cPR is reported in 26–96% of tumors compared to 57–95% with MWA. Larger tumor size and vessels larger than 3 mm adjacent to the treated tumor are the most important factors identified by previous studies associated with viable residual tumors after RFA. Correlating post-ablation radiological studies with pathological findings shows that computed tomography (CT) and magnetic resonance imaging (MRI) have low sensitivity but high specificity for detecting residual viable or recurrent hepatocellular carcinoma (HCC) tumors. There are promising recent reports combining multiprobe ablation techniques with three-dimensional treatment planning software and stereotactic-aiming instrumentation to achieve more than 90% cPR in both small and large HCC tumors. In conclusion, the reported success for achieving cPR in HCC following RFA and MWA is highly variable in different studies and decreases with increasing lesion size and unfavorable tumor characteristics. Very few studies have reported a high rate of cPR. As these studies are single-center and retrospective, they need to be further validated and reproduced in other clinical settings.

## 1. Introduction

Primary liver cancer is the fourth major cause of cancer-related mortality globally and the leading cause of cancer-related mortality in low-income countries [1]. Hepatocellular carcinoma (HCC) is a significant global health problem, representing 90% of all primary liver malignancies [2]. Since the landmark publication by Mazzaferro et al. in 1996, liver transplantation (LT) has been accepted as the most effective curative-intent therapy for patients with unresectable HCC within the predefined Milan criteria [3]. In patients for whom transplantation or resection is not an option, several local and systemic treatment options are available for the management of HCC. Thermal ablation for selected patients is the preferred treatment in many centers since it has a high potential for local tumor control [4]. 

Current European Association for the Study of Liver (EASL) guidelines for HCC management recommend thermal ablation for patients with preserved liver function, a single HCC nodule or 2–3 HCC nodules < 3 cm who are not candidates for resection or LT. Ablation is also considered as an alternative to resection for patients with very early stage HCC (solitary HCC nodule < 2 cm) [2]. Additionally, thermal ablation is a form of locoregional treatment (LRT) used for bridging or downstaging HCC patients before LT. Due to the modifications made to the Organ Procurement and Transplantation Network (OPTN) policy and concerns of overprioritizing HCC patients, these patients spend more time on the LT waitlist [5]. Thus, ablation is being utilized more often over time. 

Radiofrequency ablation (RFA) is the most widely studied thermal ablation technology applied to liver tumors. However, the risk of local tumor progression and recurrence increases in larger tumors [6] despite radiological findings of complete tumoral response. Deposition of an excessive amount of energy immediately adjacent to the RFA needle leads to tissue charring and poor energy absorption. This in turn leads to smaller coagulation volumes and residual viable tumor cells in perivascular lesions (heat-sink effect). Microwave ablation (MWA) is a newer technology developed to address the limitations of RFA.

Several previous studies have reported inconsistencies between the extent of the disease and the post-treatment response assessed radiologically with the pathological findings on the excised liver specimens after resection or liver explants at the time of LT [7,8,9]. Most of the published data regarding the efficacy of thermal ablation rely heavily on radiological studies because they are readily available for most patients. At the same time, pathological evaluation is not performed as many patients never qualify for surgery. However, studying the pathological findings on liver explants and excised liver tumors offers a unique and critically important opportunity to evaluate the efficacy of these technologies and better understand their failure. 

In this study, we reviewed the available literature regarding the application of RFA and MWA in patients with HCC, focusing on those who eventually underwent resection or LT, including associated pathological findings. A comprehensive search was performed in PubMed and Ovid Medline using combinations of the descriptors “Carcinoma, Hepatocellular/(Pathology, surgery, or therapy)”, “Liver/pathology”, “Electrocoagulation”, “Catheter ablation”, “Radiofrequency Ablation”, “Microwaves”, “Liver Transplantation” and “Pathology”. The search results were narrowed down by screening the title and abstract. Only articles published in English after 1990, given the timeframe these technologies became available, were included. Reports describing LRT without including tumor specifics treated with RFA or MWA, as well as literature without pathological correlate for the treatments, were excluded. Combined therapies using RFA or MWA with transarterial embolic methods were excluded as well since these are beyond the scope of the present discussion. Treatment parameters including the type and system for ablative technology, ablation parameters (timing, energy, number of probes), type of approach (percutaneous versus laparoscopic), as well as lesion characteristics (number and size of the tumors), response to treatment on pathology and correlation with radiological findings were abstracted and summarized.

## 2. Clinical Impact of Complete HCC Tumor Necrosis Prior to Liver Transplantation

Locoregional therapies (LRT) are vital for keeping patients with HCC on the transplant waitlist and avoiding dropouts secondary to disease progression [10]. Based on a consensus statement, LRT is recommended for all HCC patients with an expected wait time longer than six months [11]. However, the significance of the degree of tumor response to LRT and viable residual HCC tumors in liver explants and how these affect post-LT survival are essential questions that multiple studies have attempted to address. Initial studies signaled that HCC recurrence tends to happen more often in patients with more aggressive tumor features, such as vascular invasion or disease extent beyond Milan or UCSF criteria on evaluation of the liver explant irrespective of pre-LT imaging studies [12,13]. Further studies confirmed that patients who fall beyond Milan or UCSF criteria on explant histopathological evaluation have poor post-LT survival (64.0 versus 140.0 months recurrence-free survival (RFS) for patients outside versus inside Milan criteria in a cohort of 318 patients [7]). In the same study, imaging poorly correlated with explant findings since up to 20% of the patients were outside criteria at the time of LT [7]. Multiple studies have shown that achieving more than 90% necrosis [14] or complete necrosis [8,15] following LRT results in a significantly better long-term RFS after transplant compared to patients with incomplete or lower degrees of tumor necrosis. For example, in the study by Mazia et al., patients with a viable residual tumor measuring ≥2 cm, <2 cm, or no viable tumor had 5-year RFS of 61%, 81% and 95%, respectively [15]. Other studies have shown that microvascular invasion [14], the presence and amount of viable HCC following locoregional therapy [5,8,15,16] and the extent of the disease being outside UCSF or Milan criteria could independently predict HCC recurrence post-LT [14,16]. 

However, it remains unclear if the patient outcome changes after achieving a high degree of tumor necrosis before LT in patients who remain within the transplant criteria based on post-LT histopathology. In recent years, studies have been published retrospectively evaluating large multicenter cohorts of HCC liver transplant recipients. In the first study, 3601 recipients of LT from the US multicenter HCC transplant consortium included patients within the Milan criteria. The study excluded LT recipients with incidental HCC in liver explants and those beyond Milan criteria in liver explant pathology [5]. The study did not identify any difference in survival based on receiving LRT prior to LT (5-year RFS of 86% for both groups), but patients receiving LRT were more likely to be from the states with longer transplant wait times, more likely to have a greater number and diameter of tumors and were less likely to have micro- and macrovascular invasion. The study also showed that patients who, after LRT, were found to have cPR in liver explants, had superior 5-year RFS (72%) compared to patients who did not achieve cPR (69%) and patients who did not have LRT (67%) (*p*-value = 0.01) as well as a lower overall chance of HCC recurrence. Based on this, the authors investigated the effect of achieving cPR among patients receiving LRT before LT and its predicting factors in another retrospective multicenter study, including 3439 patients from 20 US transplant centers [17]. The new data showed that patients with cPR were somewhat more likely to be treated with thermal ablation as opposed to chemoembolization (28% versus 25%, *p*-value = 0.03). The 5-year HCC recurrence rate was lower (5.2% vs. 16%, *p*-value < 0.001) and 5-year RFS was higher (73% vs. 64%, *p*-value < 0.001) for patients who achieved cPR after LRT. Predictors of cPR included younger age, specific liver disease diagnosis, lower MELD score, lower AFP (alpha-fetoprotein), lower neutrophil-to-lymphocyte ratio, radiographic Milan status at the time of transplant and lower number of LRT treatments. In both studies requiring multiple sessions of LRT was associated with a poor outcome, which might be a surrogate for more aggressive or extensive tumors. 

## 3. RFA: Histological Changes in Human Tissue Following Radiofrequency Ablation

Post-RFA changes in experimental animal models are well described. In the experimental studies, coagulative necrosis immediately after the RFA was not detected since hepatocyte and sinusoidal structures were preserved or ”thermally fixed”, even though, in gross pathology, the ablation zone appears brown, surrounded by a marginal zone consisting of hyperemia and infiltrated granulocytes [18,19]. However, electron microscopy has confirmed cell death showing the destruction of mitochondria. A study of the ablation zone a few days later showed a demarcated ablation zone with coagulative necrosis in the center and preserved architecture of the hepatic cords and sinusoids in the periphery surrounded by fibrosis [18]. Similarly, electron microscopy and TUNEL staining have confirmed irreversible cell injury by showing destroyed nuclei and mitochondria and increased apoptotic activity in the peripheral zone [19,20]. Additionally, these preclinical experiments confirmed the presence of intact vessel walls and the survival of hepatocytes adjacent to them [18], in line with results from HCC ablation outcomes and the association of large peritumoral blood vessels with incomplete PR following RFA [6].

As in observations from preclinical experiments, post-RFA changes in human tissue did not meet the criteria for coagulative necrosis in the first three days. However, cell viability markers were absent on histochemical evaluation suggesting irreversible cell damage [21,22]. In subacute (1–4 weeks) and chronic (more than 4 weeks) stages, the coagulum in human liver tissue specimens shows complete coagulative necrosis in the center with thermal fixation in the periphery surrounded by a narrow hypocellular fibrous rim with a giant cell-type reaction with interstitial hyperemia and hemorrhage outside this rim [21,22,23]. Studies have also shown that the evaluation of samples with more advanced staining techniques such as TUNEL increases sensitivity in the detection of complete tissue necrosis [24]. 

## 4. RFA: Pathological Correlation 

Goldberg et al. performed a pilot study in 2000 by examining post-RFA changes in patients with HCC or colorectal cancer metastases in the liver either excised immediately after intraoperative ablation or 3–7 days following percutaneous ablation. They observed that although coagulative necrosis cannot be diagnosed immediately after intraoperative ablation, ablated tissue shows changes in the mitochondrial and cytosolic enzyme activity suggesting irreversible cell damage [21]. On the other hand, samples excised at least three days after ablation showed coagulative necrosis correlating with nonenhancing areas on cross-sectional images obtained immediately after the RFA. 

Since this pilot experiment correlating the post-ablation cross-sectional imaging with actual histologic changes in human liver tissue in vivo, multiple studies have evaluated the tumor response following thermal ablation on either excised tumors or explanted livers during transplantation. Most of these studies have been on HCC since thermal ablation is used as bridge therapy for HCC patients to liver transplantation. This enables a comprehensive evaluation of response to local treatment on the explant pathology. Table 1 summarizes the available literature reporting the tumor response and rate of achieving cPR for HCC tumors following RFA. 

The rate of achieving cPR was highly variable among different studies and secondary to the patient, technique/experience and technology-related factors. The rate of achieving cPR was reported to be as high as 75% [6,23,24,25,31] but as low as 8% in some other studies [24]. It should be considered that differences in the wait times before liver transplantation and variations in liver histological examination methodology among different pathology departments complicate interstudy comparison. For example, Mazzaferro et al. [27] observed that increasing wait time following RFA increases the chance of finding viable residual tumor in liver explants. It has also been shown that using more advanced staining techniques such as TUNEL increases detection of cPR [30]. 

More recent studies have tried to address important questions about RFA and compared different RFA technologies in achieving cPR based on explant evaluation. Seror et al. [33] compared the efficacy of internally cooled (Covidien, Mansfield, MA, USA) or internally perfused (HITT Integra, Tubingen, Germany) RFA electrodes in monopolar mode versus the internally cooled electrodes in the multipolar mode in a total of 35 patients with 59 HCC nodules. Patients had similar baseline characteristics, including age, gender, etiology, Child–Pugh Class, AFP level and nodule size. Based on the outcome of cPR in liver explants, multipolar technique using internally cooled RFA electrodes (26/29, 90%) outperformed the monopolar technique using either internally cooled (8/17, 47%) or internally perfused electrodes (6/13, 46%).

Along with the improvement of RFA technology, the invention of targeting devices and development of planning software for precise needle placement using real-time imaging data, increasing knowledge about the shortcomings of RFA technology, has led to improved success rates. Bale et al. [35] reported on a study of a large cohort including 96 patients with 188 HCC nodules measuring 1–8 cm in diameter (mean 25 mm) with liver explant evaluation following stereotactic RFA bridging therapy to liver transplantation with a reported success rate of 97% (183/188) in achieving cPR, including a 96% (50/52 tumors) success rate in tumors larger than 3 cm in diameter. Stereotactic RFA combines a rapid-switching, multiple-electrode RFA system with internally cooled probes (Covidien, Burlington, MA, USA) with high precision probe insertion using a combination of planning software and a percutaneous aiming device. Although the numbers are promising, the data resulted from a single-center experience and it is thus unknown if similar results can be obtained at other institutions. 

## 5. RFA: Factors Associated with the Incomplete Pathological Response 

Despite differences among studies, most available data are consistent in that larger lesions tend to respond poorly to RFA. A diameter of 3 cm has been proposed as a cut-off. Mazzaferro et al. observed that although 55% of the treated HCC lesions in their study had cPR on the explants, the smaller lesions tend to respond better (63% cPR for HCC tumors ≤ 3 cm) and tumor size was the only prognostic factor related to the success of RFA [27]. Similarly, in the studies by Lu et al., lesions 3 cm or less in diameter had 80% cPR compared to only 39% for larger tumors (mean diameter of lesions with cPR was 2.0 cm compared to 3.1 cm for tumors with viable residual tumor) [6,29]. Several other studies have confirmed these findings [28,34] and, more recently, in the series reported by Serra et al., cPR was achieved in 77%, 55% and 31% of ≤2 cm, 2–3 cm and >3 cm HCC nodules, respectively [34].

The other important factor that lowers cPR rates in ablation is perfusion-mediated tissue cooling or heat-sink effect, as shown by experimental studies [36]. This is a known problem for ablating tumors that are close to or abutting large vessels. This is underscored by the results from a study by Lu et al. cPR was achieved in only 47% (7/15 tumors) of the tumors when there was a vessel 3 mm or larger abutting the tumor or entering it, compared to 88% (28/32 tumors) for nonperivascular lesions (*p*-value = 0.009) [6].

## 6. MWA: History and Comparative Aspects

Microwave ablation is a relatively newer technology originally used to help achieve intraoperative hemostasis [37]. MWA was later translated as a thermal ablation device to address the shortcomings of RFA technology, such as the long operative time, limited coagulum size and, most importantly, the heat-sink effect resulting in preserving viable tumor cells adjacent to larger blood vessels [38]. Although MWA is theoretically superior to RFA, several prior studies comparing the two modalities have failed to demonstrate any clinical benefit of MWA over RFA [4,39,40]. For example, a randomized clinical trial evaluating 72 patients who were randomly assigned to either MWA or RFA failed to show any difference in achieving complete response or local recurrence between the modalities [4]. 

## 7. MWA: Histological Changes in Human Liver

Macroscopic and microscopic changes following MWA have been studied extensively in experimental models. In gross pathology examination, the zone of coagulation consists of a pale center with areas of cavitation seen more frequently closer to the site of MWA antenna resulting from tissue fluid heating and bubble formation. The pale white center of the ablation zone is immediately surrounded by a brown thin capsule peripherally [41,42]. At the microscopic level, there is a thin layer of spongy and severely damaged cells at the center of the ablation and, beyond this area, three distinct concentric zones have been described somewhat similar to the rings of a target. Immediately beyond the spongy center, there are deformed cells with damaged cellular membranes, shrunken and destroyed internal organelles and loss of enzymatic activity (zone A). Zone B consists of swollen hepatocytes with the damaged cellular membrane and eosinophilic cytoplasm (coagulative necrosis) and, finally, morphologically normal-appearing hepatocytes within a hemorrhagic stroma are present at the margins (zone C) [43,44,45]. Amorphous material, debris and damaged red blood cells within sinusoids and edema within portal triads and septa are also present in zones A and B compared to some degree of congestion in zone C [46]. 

## 8. MWA: Pathological Correlation 

Given the more recent commercialization of microwave technology, fewer data are available correlating the histological findings of excised or explanted livers to assess cPR from MWA. In 1998, Dong et al. [47] reported one of the first experiences with MWA in the liver for a heterogeneous group of patients suffering from either HCC or metastatic liver disease. From 41 patients with HCC, they obtained post-treatment biopsies for 19 patients with no viable tumor seen in 95% (18/19) of the biopsies. However, the limitations of a single biopsy precluded a comprehensive pathological evaluation. In 2003, Yamashiki et al. [48] examined 18 tumors in 15 liver explants following laparoscopic MWA and reported a cPR in 15/18 tumors. In 2 out of 18 tumors, the residual disease was secondary to satellite nodules, in one case adjacent to the zone of coagulation and in the other one in the same segment. In 2003, Dong et al. [49] published the results of percutaneous MWA in 234 patients with 339 HCC nodules. They reported the treatment response rate using a biopsy or excision within three months of the ablation in 200 tumors with a response rate of about 93%, with a local recurrence in 17/234 patients (7%). Recurrences were located away from the zone of coagulation in another 23% of the patients and the overall survival of the patients was 72% with a mean follow-up period of 28 months. In 2005, Meredith et al. [50] performed a clinical study of five patients with six liver tumors aiming at partial ablation of the tumors using a dual-loop probe system and showed that the dual loop system is capable of performing precise MWA in human liver tissue. Later, Simon et al. [51] examined the feasibility of using multiple single-probe MWA in the liver for the treatment of large HCC and metastatic disease (up to 57 mm in diameter) followed by tumor excision and demonstrated coagulation necrosis even surrounding large blood vessels (>3 mm), an area of failure for RFA. In 2006, Yu et al. [52] compared single straight versus triple straight or loop antennas in nine patients with resectable HCCs and concluded that use of a triple-loop configuration microwave device results in the most uniform spherical ablation, although the size of ablation was comparable to a triple straight antenna configuration. NADH diaphorase staining confirmed complete necrosis of the cells even in the transition zone observed between the coagulated and viable area on the samples’ gross examination. However, they did not report the rate of achieving complete tumor necrosis in their samples. Kuang et al. [53] reported the clinical outcomes of MWA using an internally cooled probe in 133 tumors in 90 patients (74/90 patients had HCC) in HCC and liver metastases with a mean diameter of 2.7 cm (Range: 0.8–8.0 cm) with complete ablation rates of 94%, 91% and 92% and local tumor progression rates of 6%, 6% and 0% (based on computed tomography (CT) or magnetic resonance imaging (MRI)) for lesions ≤ 3 cm, 3.1–5.0 cm and 5.1–8.0 cm, respectively, during a mean follow up of 17.4 months but the study lacked pathological correlation. In 2011, Zanus et al. reported the explant histological evaluation results of six patients with HCC ranging from 25–50 mm in diameter with 100% cPR. Although this was a small study of relatively short duration, there was no HCC-related mortality within the follow-up period of more than 12 months [54]. Baimas-George et al. in 2020 reported higher cPR rates after MWA (57%) for bridging HCC patients to LT compared to TACE (13%) or combined MWA/TACE (29%), which was associated with better overall survival. There was also less viable residual tumor in liver explants after MWA (17.2% after MWA versus 48.7% after TACE and 18.6% after combined MWA/TACE) [55]. Table 2 provides a summary of the available studies cited here. 

## 9. Radiological and Histological Correlation

Post-RFA cross-sectional imaging with computed tomography or magnetic resonance imaging closely correlates with the histologic description of the alterations in the zone of coagulation. Imaging studies have shown an area without contrast enhancement immediately after ablation [21] followed by the appearance of an enhancing rim surrounding the treatment area at around day three, which resolves over time [22], likely correlating with the fibrotic rim and hyperemia in pathology [23]. The zone of coagulation then starts shrinking over time on imaging [22] and an increase in the size of the ablated area after the subacute phase should be considered suspicious for tumor recurrence. 

Despite this close visual correlation, previous studies have confirmed disparities between cross-sectional imaging and explant pathology resulting in understaging the extent of HCC disease before LT [7], as well as the response to locoregional therapy [9]. Ecker et al. showed that MRI could lead to understaging in about 20% of patients, negatively affecting the post-transplant course overall, as well as RFS [7]. In a blinded study, radiologists and pathologists independently evaluated HCC recurrence in CT/MRI or liver explants following local therapy with either RFA or TACE, showing a 40% sensitivity and 100% specificity for cross-sectional imaging predicting viable residual tumor [9]. The study also defined three different patterns for viable/recurrent HCC within the coagulation volume, including discontinuous rim, nodular and solid. In their small cohort imaging studies, radiologists detected solid pattern cases but missed all the discontinuous rim pattern recurrence. 

Additionally, evaluating the liver explants in HCC patients has resulted in the unexpected discovery of satellite nodules around the target lesion and remote tumors not diagnosed in pretransplant imaging. The presence of satellite nodules is reported in 8–57% of treated tumors [6,12,25,27,32] and appears to be associated with larger HCC tumors [27]. New remote HCC nodules have been reported in 13–50% of the explanted livers depending on the study [12,13,24,25,26,27,31]. Several studies have evaluated the sensitivity and specificity of cross-sectional imaging studies for identifying viable residual tumors in the treatment zone [16,32,35,56]. The data are summarized in Table 3. Overall, it appears that imaging findings of recurrence or viable residual tumor in cross-sectional imaging are very accurate, with specificity ranging from 80–100% in most studies. Simultaneously, negative imaging studies poorly predict the absence of viable tumors on the liver explant’s histopathologic evaluation after liver transplantation, resulting in below 50% sensitivity as reported by several studies.

Similar to RFA, imaging following MWA shows a nonenhancing area corresponding to the zone of coagulation. The majority of lesions develop an enhancing rim after about one week following treatment, which disappears over time, and failure to do so or an increase over time should be considered a sign of a residual or recurrent tumor [47,48]. There are few studies available that would allow direct radiological-pathological correlation after MWA. These studies recommended low sensitivity and positive predictive value (PPV) and higher specificity and negative predictive value (NPV) to detect viable residual tumor after ablation [48,54]. 

## 10. Conclusions

The reported success for achieving cPR in HCC following RFA and MWA is highly variable in different studies and tends to decrease with increasing lesion size and unfavorable tumor characteristics. Published outcomes must be interpreted in context, as the studies include a relatively broad time span and ablation technologies continue to evolve. Despite promising data from a limited number of studies that have achieved a high rate of cPR by creating large volumes of coagulation using multiple probes and advanced computational technology, the data is limited, single-center and retrospective and needs to be further validated and reproduced in other clinical settings. Given the adverse outcomes associated with an incomplete pathologic response, there remain many opportunities to improve ablation technology. 

## Figures and Tables

**Table 1:** Comparison of the rate of complete response following radiofrequency ablation (RFA) for the local control of hepatocellular carcinoma (HCC) based on histopathological evaluation of the excised tumors or liver explants at the time of transplantation.

Reference	Type of Ablation	Type of Tissue	Number of Patients	Tumor Characteristics			cPR	pPR ^†^	Vascular Invasion	Satellite Nodules	Remote or Additional Nodules
				Number	Mean Diameter (mm)	Range (mm)					
Goldberg et al. [21]	Perc Single IC * Radionics	Excised	1	1	34	NA	0/1 (0%)	1/1(100%)		NA	NA
Pulvirenti et al. [25]	Perc Single EP ** RITA Medical	Liver explant	14	16	35	17–60	12/16 (75%)	4/16 (25%)		8/14 (57%)	3/14 (36%)
Fontana et al. [13]	Perc Single/Multiple EP RITA Medical	Liver explant	14	14	35	20–51	4/14 (29%)	10/14 (71%)	3/14 vascular invasion, 2 developed post LT recurrence		7/14 (50%)
Morimoto et al. [22]	Perc RFA Single/overlapping EP Leveen Needle RadioTherapeutics Co.	Excised	5	6	20	14–30	4/6 (66%)	2/6 (33%)			
Coad et al. [23].	Perc or Surg Single EP RITA Medical	Liver explant	3	4	25	20–42	3/4 (75%)	1/4 (25%)			
Wong et al. [26]	Perc RFA (exact method not included)	Liver explant	4	5	22	10–45	3/5 (60%)	1/5 (20%)	1/5 (20%)		2/15 (13%)
Mazzaferro et al. [27]	Perc or Surg RFA EP RITA Medical or ICP Radionics	Liver explant	50	60	28	10–80	33/60 (55%)	27/60 (45%)		14/50 (28%)	18/50 (36%)
Pompili et al. [28]	Perc Single/overlapping EP RITA Medical or ICP Radionics	Liver explant	25	30	26	10–65	14/30 (47%)	16/30 (53%)			
Lu et al. [29]	Perc RFA Single/overlapping EP Leveen (RadioTherapeutics Co.), EP RITA Medical or ICP Radionics	Liver explant	42	64	25	4–57	45/64 (70%)	19/64 (30%)			
Lu et al. [6]	Perc RFA Single/overlapping EP Leveen (RadioTherapeutics Co.), EP RITA Medical or ICP Radionics	Liver explant	24	47	23	4–55	35/47 (74%)	12/47 (26%)		2/24 (8%) included in the pPR	
Martin et al. [24]	Surg or Perc Single/overlapping EP RITA Medical	Liver explant	35	38	25	10–50	28/38 (74%) (more than 90% necrosis)	10/38 (26%)			8/35 (23%)
Netto et al. [30]	Surg or Perc Single/overlapping EP RITA Medical	Liver explant	35	36	25	17–50	14/36 (39%) based on TUNEL	22/36 (61%)			
Brillet et al. [31]	Perc Single/overlapping EP RITA Medical	Liver explant	13	16	24	10–50	12/16 tumors (75%)		4/13 (31%) vascular invasion	7/13 (54%)	7/13 (54%)
Rodrigues-Sanjuan et al. [32]	Perc Single/overlapping EP Leveen (RadioTherapeutics Co.), EP ICP Radionics	Liver explant	28	30	31	15–60	14/30 (47%)	16/30 (53%)			
Marin et al. [12]	Perc or Surg Single/overlapping EP Leveen (RadioTherapeutics Co.) or EP RITA Medical	Liver explant	10	12	32	22–48	1/12 (8%)	11/12 (92%)		2/12 (17%)	3/10 (30%) for a total of 5 nodules
Seror et al. [33]	Perc Single/overlapping Monopolar ICP (Covidien) vs. Monopolar IP *** (HITT Integra) vs. Multipolar ICP (Prosurge)	Liver explant	11 8 16	17 13 29	25 22 21	10–35 10–40 15–55	8/17 (47%) 6/13 (46%) 26/29 (90%)				
Serra et al. [34]	Perc Single/overlapping ICP (Radionics)	Liver explant	78	125	20	16–26	<2 cm 40/52 (76.9%) 2–3 cm 33/60 (55%) >3 cm 4/13 (30.8%)	<2 cm 12/52(23.1%) 2–3 cm 27/60(45%) >3 cm 9/13(69.2%)			
Bale et al. [35]	Perc Overlapping stereotactic multiprobe ICP (Covidien)	Liver explant	96	188	25	10–80	Overall 183/188 (97.3%) >3 cm 50/52 (96.2%)	Overall 5/188 (2.7%)			

*: IC: internally cooled, **: EP: expandable probe, ***: IP: Internally perfused, ^†^: pPR: partial pathological response.

**Table 2:** Comparison of the rate of complete response following microwave ablation (MWA) for the local control of HCC based on histopathological evaluation of the excised tumors or liver explants at the time of transplantation.

Reference	Year	Approach	Device	Tissue	Number of Patients	Tumor Characteristics			cPR	pPR	Satellite Nodules	Remote Tumors
						Number of Tumors	Mean Tumor Size (mm)	Range (mm)				
Dong et al. [47]	1998	Perc	2450 MHZ, 10–60 W, 240–300 s Single straight	Biopsy	41	41	43	18–87	18/19 (95%)	1/19 (5%)	NA	NA
Yamashiki et al. [48]	2003	Surg	2450 MHZ, 80 W, 60 s Single straight	Explant	15	18	34	12–50	15/18 (83%)	3/18 (17%)	2/15 (13%)	
Dong et al. [49]	2003	Perc	2450 MHZ, 75 W, 180–300 s Multiple straight	Biopsy/excision (within three months)	234	339	41	12–80	185/200 (93%)	15/200 (7%)	NA	NA
Meredith et al. [50]	2005	Surg	2450 MHZ, 60 W, 300–420 s Dual loop	Excised	5	6	NA	NA	NA	NA	NA	NA
Simon et al. [51]	2006	Surg	915 MHZ, 45 W, 600 s Triple straight	Excised	4	4	44	20–57	NA	NA	NA	NA
Yu et al. [52]	2006	Surg	915 MHZ, 60 W, 300–600 s Single/triple straight or triple loop	Excised	9	9	42	29–60	NA	NA	NA	NA
Zanus et al. [54]	2011	Surg/Perc	2450 MHZ, 30–60 W, 240–600 s Straight internally perfused	Explant	6	6	34.5	25–50	100%	0%		
Baimas-George [55]	2020	NM	NM (not mentioned)	Explant	14	32	31.5	18–70	8/14 (57%)	6/14 (43%)		

**Table 3:** Cross-sectional imaging correlation with histopathological findings in liver explants after RFA or MWA.

Reference	Type of Imaging	Type of Thermal Ablation	Criteria	Sensitivity	Specificity	PPV *	NPV **
Mazzaferro et al. [27]	CT	RFA	Complete necrosis in treated HCC nodule	64%	73%		
Lu et al. [6]	CT/MRI	RFA	Complete necrosis in treated HCC nodule	36%	100%	100%	83%
Pulvirenti et al. [25]	CT	RFA	Complete necrosis in treated HCC nodule	100%	100%	100%	100%
			Overall viable HCC in the explant	45%	100%	100%	40%
Brillet et al. [31]	CT/MRI	RFA	Complete necrosis in treated HCC nodule	75%	83.3%	60%	90.9%
			Overall viable HCC in the explant	62.5%	100%	100%	62.5%
Rodrigues-Sanjuan et al. [32]	CT	RFA	Complete necrosis in treated HCC nodule	50%	100%		
Serra et al. [34]	CT/MRI	RFA	Complete necrosis in treated HCC nodule	93.2%	51.2%	77.3%	80.8%
Bale et al. [35]	CT/MRI	RFA	Complete necrosis in treated HCC nodule	40%	100%	100%	98.4%
Yamashiki et al. [48]	CT	MWA	Complete necrosis in treated HCC nodule	66.7%	93.8%	66.7%	93.8%
Zanus et al. [54]	CT	MWA	Complete necrosis in treated HCC nodule		83.3%		100%
Baimas-George [55]	CT	MWA	Complete necrosis in treated HCC nodule	50%	86%	75%	67%

*: PPV: positive predictive value, **: NPV: negative predictive value.
